# Is working in later life good for your health? A systematic review of health outcomes resulting from extended working lives

**DOI:** 10.1186/s12889-021-11423-2

**Published:** 2021-07-09

**Authors:** Susan Baxter, Lindsay Blank, Anna Cantrell, Elizabeth Goyder

**Affiliations:** School of Health and Related Research, University of Sheffield, Regent Court, Regent Street, Sheffield, S14DA UK

**Keywords:** Extending working, Retirement, Health, Systematic review, Employment, Older workers

## Abstract

**Background:**

Work, rather than unemployment, is recognised as being good for health, but there may be an age when the benefits are outweighed by adverse impacts. As countries around the world increase their typical retirement age, the potential effect on population health and health inequalities requires scrutiny.

**Methods:**

We carried out a systematic review of literature published since 2011 from developed countries on the health effects of employment in those over 64 years of age. We completed a narrative synthesis and used harvest plots to map the direction and volume of evidence for the outcomes reported. We followed the Preferred Reporting Items for Systematic Reviews (PRISMA) checklist in our methods and reporting.

**Results:**

We identified seventeen relevant studies, which were of cohort or cross-sectional design. The results indicate evidence of beneficial or neutral effects from extended working on overall health status and physical health for many employees, and mixed effects on mental health. The benefits reported however, are most likely to be for males, those working part-time or reducing to part-time, and employees in jobs which are not low quality or low reward.

**Conclusions:**

Extending working life (particularly part time) may have benefits or a neutral effect for some, but adverse effects for others in high demand or low reward jobs. There is the potential for widening health inequalities between those who can choose to reduce their working hours, and those who need to continue working full time for financial reasons. There is a lack of evidence for effects on quality of life, and a dearth of interventions enabling older workers to extend their healthy working life.

**Supplementary Information:**

The online version contains supplementary material available at 10.1186/s12889-021-11423-2.

## Background

There has been increasing policy focus on retaining older workers in the workforce [1]. The rationale cites the fact that whilst retirement age has been getting higher since the 1990s, it has not kept up with increasing life expectancy [2]. There are also concerns around the globe regarding addressing the increasing “old age dependency ratio” [3].

While the evidence is clear that generally work rather than involuntary unemployment is good for health (particularly mental health) [4], there may be an age when the benefits are outweighed by adverse impacts. The removal of a default retirement age in many countries potentially gives employees more choice regarding when to retire [3, 5, 6]. However, around a third of those working beyond state pension age report that they have remained in employment due to financial necessity [6]. There is the potential for policies which extend working lives, to adversely impact on health inequalities in older age, and act differentially in population sub-groups [7, 8].

Requiring older workers to remain in employment because of increasing life expectancy may lead to a perception of victimisation, as they perceive that they are paying for those who have already retired at a younger age than them [7]. It has been reported that there can be a sense of unfairness from extending working age amongst current workers, which can negatively affect production and job satisfaction [7]. While the agenda to drive extended working has principally focused on the economic needs and benefits, it is known that employees grapple with both “push” and “pull” factors in their decision-making about when to retire [9, 10]. The value of mental stimulation and a need for pension security in older age tend to pull employees to remain in work, while other commitments such as family, leisure activities and having financial security push workers towards retirement [7]. Authors have identified that actions by managers to support successful ageing in the workplace can determine intentions to stay [11].

While the case for extended working lives in terms of productivity and avoiding labour shortages [12] and maximising individual pension income has been established [9], the link between extended employment and health outcomes is less well understood. It is important to examine the health outcomes associated with increased retention of older workers (both positive and negative) to inform policy and guidance, and also so interventions can maximise any health benefits of employment, and mitigate any potential harms.

The overall aim of this review was to synthesise existing research evidence on health outcomes resulting from the extension of working lives, and to evaluate the effectiveness of interventions which have the aim of optimising healthy extended working.

## Methods

### Search strategy

Searches were conducted in the Medline, Applied Social Sciences Index and Abstracts and PsycINFO databases. The searches were limited to papers in English, published from 2011 to June 2020. The full search strategy in an example database is provided as a supplementary file. We searched for citations of key included papers and authors, screened reference lists, and scrutinised relevant websites for grey literature (see supplementary files).

### Inclusion criteria

We included any type of study which reported empirical data. Our population of interest was workers in paid employment beyond typical or statutory retirement age. We used age 64 or over as the criteria, as this is the average effective age of labour market exit across developed countries [13]. We included studies which compared any health or health-related outcome in those retired to those continuing to work. Studies which only referred to “older workers” or “retirees” where we could not distinguish those above aged 64 from younger participants were excluded. We included relevant literature from developed countries (members of the Organisation for Economic Co-operation and Development) published since 2011. This date was chosen as the results were intended to be directly relevant to our UK funder and this is the date of the removal of the default retirement age in the UK.

### Quality appraisal

We used checklists appropriate to each of the study designs. These checklists were from the Critical Appraisal Skills Programme suit of appraisal tools [14]. We considered the relative weight that could be attached to individual research studies, and the robustness of the body of evidence as a whole in terms of volume, consistency and quality [15].

### Screening and data extraction process

Search results were downloaded to a reference manager database (Endnote version X9) and screened by one reviewer, with 20% checked by a second reviewer. Potentially relevant citations were retrieved for review at full paper level. We extracted author/year; study design; population; outcomes; findings; and main conclusions for studies meeting our inclusion criteria. Data extraction was carried out by a team of three reviewers, with all studies extracted by one reviewer and checked by a second.

### Synthesis method

The findings were synthesised narratively, and characteristics of the literature were tabulated to provide a summary overview. We used Harvest Plots [16, 17] to summarise the evidence relating to adverse, neutral or positive effects of extended working on reported outcomes.

## Results

**Fig. 1:**
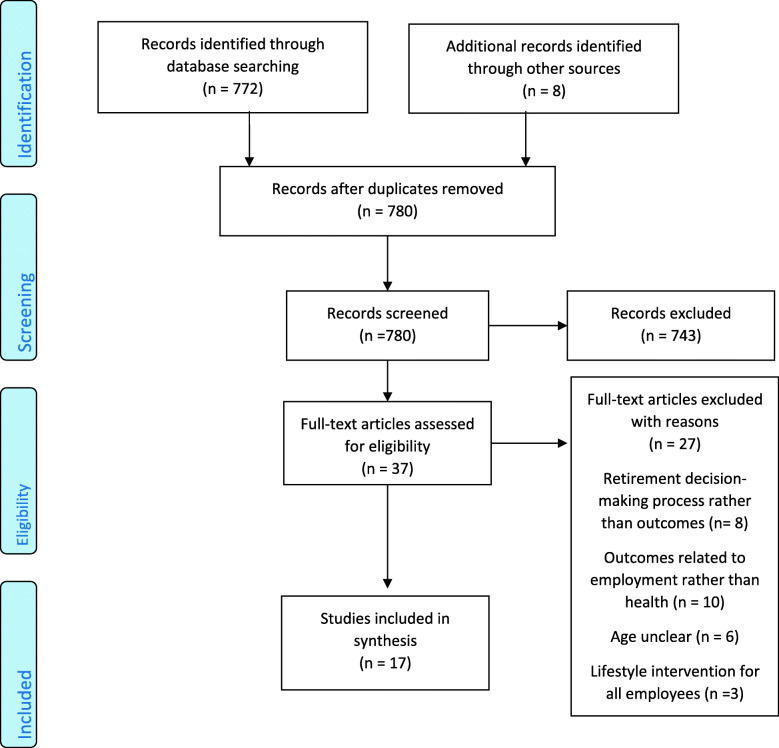
PRISMA diagram illustrating the selection process

The searches generated 772 records, of which 36 were retrieved as full papers. Of these, nine were found to meet the inclusion criteria. An additional four papers were identified from checking reference lists of included studies, and a further four were identified via citation searching. See Fig. 1 for a diagram illustrating the selection process as recommended by the PRISMA guidelines [18]. The list of papers excluded at full paper screening and extraction table are provided as an additional file.

### Characteristics of the literature

The evidence is from six individual countries (see Table 1), and two sources included data from multiple countries. Japan provided the largest volume of studies, followed by the United Kingdom (UK). The sources are therefore representative of populations with varying healthcare systems and employment practices.

**Table 1:** Summary of the studies

First author, year Location	Population	Study design	Health, physical health, mental health and other outcomes reported in the study
Anxo 2019 Sweden	Age 65+ retirees, *n* = 8022	Longitudinal routine data	Health - self-assessed health Physical health - self-reported fitness Mental health - depressive symptoms, well-being
Ardito 2016 Italy	Aged 68–70, *n* = 94,52. Male retired (defined as receiving an occupational pension) and those still in employment after pension age	Retrospective analysis of routine data	Health - cardio vascular disease incidence
Blok 2011 Netherlands	Older than 64	Review	Mental health - effect on sleep Other - accidents and work performance
Carmichael 2013 UK	Aged 50–68 years, *n* = 56. Just over half male, 30% retired, 39% in paid employment	Qualitative	Other - perceived relationships between health and employment Other - labour market participation
Carrino 2018 Italy (Data from UK)	Women aged 60–64 average age 62.5, *n* = 3452	Retrospective analysis of survey data	Other - effects of pension reform on sickness, caring and incomeself-reported employment status
Di Gessa 2017 UK	Men 65–74, women 60–69, *n* = 2039	Retrospective analysis of survey data	Health - self-report of doctor diagnosed heart disease/stroke, report of long-standing illness Physical health - grip strength, mobility limitations Mental health - depression, somatic health, sleep disturbance Other - employment history
Di Gessa 2018 UK	Men aged 65–74, women 60–69 at baseline. 56% female, *n* = 2502 (longitudinal analysis) 1823 (cross sectional analysis)	Retrospective analysis of survey data	Physical health - activities of daily living Mental health - illness, depression, self-realisation and pleasure Other - control, autonomy, social relationships and contacts, employment status
Farrow 2012 UK	60+	Systematic Review	Other - injuries and accidents, sickness absence
Fujiwara 2016 Japan	Aged 65–84 years, *n* = 981	8-year longitudinal study	Health - medical history, smoking status Physical health - activities of daily living, exercise habits, walking speed Mental health - life satisfaction Other - working status
Kajitani 2011 Japan	Age 60+ male, *n* = 2032	Estimation model	Health - self-assessed health status, presence of disease, life expectancy Physical health – self-reported physical limitations, nutrition
Kalousova 2015 European + US data	Older workers mean age 55 at baseline, male, *n* = 2475	Cohort study	Physical health – self-reported frailty, handgrip, walking speed
McDonough 2017 UK authors using US data	Aged 52–69 and early 70s, *n* = 6522	Cohort study	Health - self-rated health Physical health - functional limitations
Minami 2015 Japan	Age 65+ (mean 73.4), *n* = 1768	Cohort study	Health - self-rated healthMental health – self-rated mental health Other - higher-level functional capacity
Morelock 2017 US	Older workers in healthcare, *n* = 437	Time and place management intervention	Other – workability (ability to carry out a job)
Okamoto 2018 Japan	Aged 60 or older males, *n* = 1288	Cohort study	Health – mortality, cognitive decline, self-reported symptoms of stroke, diabetes
Potocnik 2013 European dataset	Average age 69.79 years. Retirees and older employees, *n* = 2813 retirees and *n* = 1372 older employees	Cohort study	Physical health - engaging in sport or voluntary activities Mental health - depression, quality of life
Stenholm 2014 Finland (data from US)	Aged 65–85 years, *n =* 17,844	Cohort study	Physical health - self-reported physical functioning
Tomioka 2018 Japan	Aged over 65 years, *n* = 6417	Cohort study Postal questionnaire	Health – report of cognitive decline Physical health - care needs, activities of daily living
Welsh 2016 Australia	Aged 50–59 years at baseline, *n* = 836 older workers	Cohort study	Health – self-reported health Physical health - self-rated physical activity Mental health – self rated mental health

The most common design was a cohort study. Follow up periods were from one year to nine years. There was one randomised controlled trial of an intervention, and two other relevant systematic reviews. The completed quality appraisal for each study is provided as supplementary data. In general the studies achieved most items of the quality ratings, with no serious concerns regarding bias or confounding factors. We report the study design as an indicator of quality in the synthesis. The two other reviews we identified were of shift work tolerance [19] and injuries and accidents amongst older workers [20]. The majority of studies included both male and female employees, with two having only male participants [21, 22] and one researching only female employees [23].

### Associations between extended working life and health and health-related outcomes

**Fig. 2:**
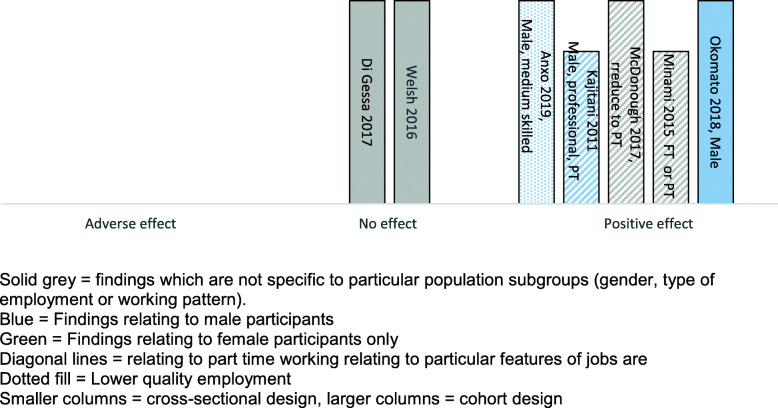
Harvest *plot summarising results of studies reporting health outcomes*

**Fig. 3:**
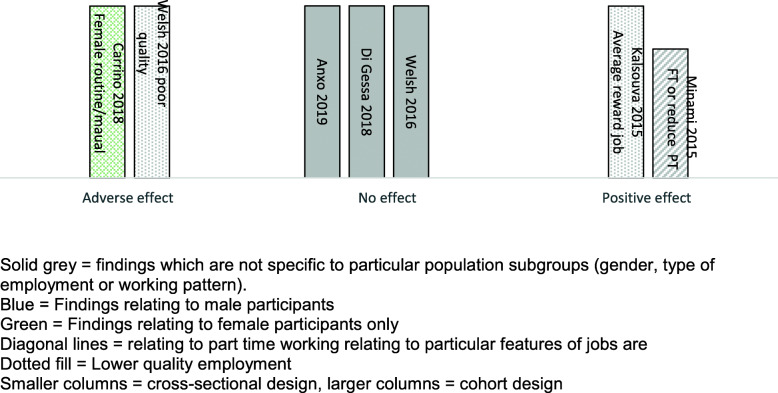
Harvest *plot summarising results of studies reporting mental health outcomes*

**Fig. 4:**
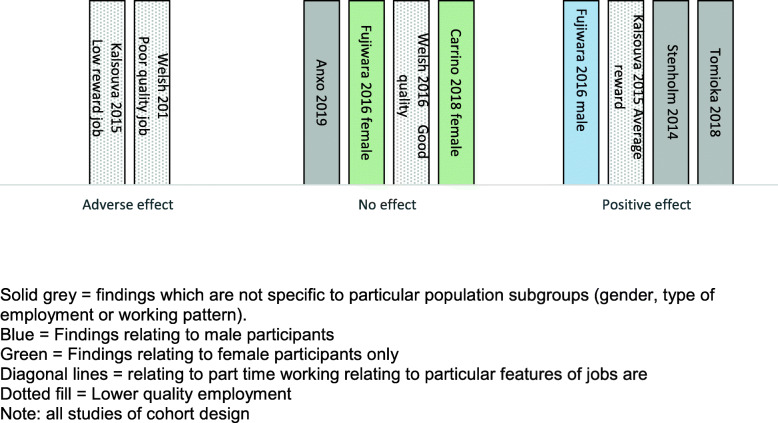
Harvest plot summarising the results of studies reporting physical health-related outcomes

Sixteen studies provided data on the effects of extended working life on health. Many of the studies provide complex findings, with differential effects reported for sub-groups of employees, for different patterns of working, or for different outcomes. We have therefore used Harvest plot methods to provide a visual summary of the volume and direction of evidence (see Figs. 2, 3, and 4). Where there are varying findings within a single study, these have been represented by multiple entries, therefore it is important to note that the plots do not always provide a simple count of studies.

#### The effect of extended working life on overall health

Seven studies measured overall health effects (described as self-assessed health, self-rated health status, somatic health, or general health) using self-reported questionnaires. By volume there are greater indications of positive rather than neutral or negative effects of extended working life on health (Fig. 2). Five studies reported positive effects (all but one of these for particular sub-groups of workers), two neutral effects, and none adverse effects.

Of the five studies reporting positive effects, two of them were carried out in Japan with male participants only [21, 22], and in a further study (carried out in Sweden) the evidence of positive effects relates to males only [24]. In one study [22], in addition to a positive effect on self-reported health, being employed reduced susceptibility to stroke (14% versus 20% *p* = 0.007), although had no effect on the incidence of diabetes (*p* = 0.878). A cohort study of Japanese adults aged over 65 [19] found that workers had significantly better self-rated health compared to non-workers (*p* < 0.001).

An evaluation using linear probability modelling estimated that for males (not females), having work experience after the retirement age of 65 would on average increase the probability of reporting better health during retirement by about 14% (adjusted to 11% when controlled for demographics and socio-economic characteristics). Even after controlling for pre-retirement health there remained an estimated effect of 0.068 (*p* < 0.01) [24]. However, calculations estimated that the health advantage would be lost after 6 years of retirement.

Some studies highlighted that reducing to part-time work in older age was associated with positive health outcomes. A cohort study from the US indicated that those who had reduced to part time working around age 62 or 65 were less likely than almost all other groups (except women working part time throughout) to report poor health in their early 70s (men odds ratio (OR) OR 0.49 *p* < 0.01; women OR 0.29 *p* < 0.001) [25].

The studies reporting neutral health effects of extended working were a cohort study from Australia [26], and a retrospective analysis of UK national survey data [27]. In the first, analysis indicated no significant difference in self-rated health between those who were working and those who voluntarily retired (*p* = 0.825). In the second study, initial analyses had suggested that men and women in paid work were more likely to report better health at follow-up than those who were not working however, once baseline socioeconomic and health and labour market histories were accounted for, the health benefits of working beyond statutory pension age were no longer significant.

#### The effect of extended working on mental health

Five studies reported mental health outcomes associated with extended working. The volume and direction of evidence for this outcome was mixed, with one study finding a positive effect, three a neutral effect, and two an adverse effect for some workers (see Fig. 3).

The single study reporting a positive effect on mental health was a cohort study of Japanese adults aged over 65 [28]. Those working either full time or part time work had significantly better mental health (as measured by the Geriatric Depression Scale), compared to those who had retired.

In one study [27] initial analysis indicated that respondents in paid work beyond statutory pension age were less likely to be depressed or report sleep disturbance, but in the fully adjusted results the benefits were no longer significant. Other reports [24] similarly found no statistically significant effect of working longer on self-reported depressive symptoms/well-being or mental health between those who were working and those who were voluntarily retired.

An adverse outcome for females who work for longer was found by one UK study which examined the effects of a change in state pension age for women, with poorer mental health (− 3%), and higher depression scores (+ 10%) [23]. Also, those in routine manual occupations were more negatively affected than those in professional occupations.

#### The effect of extended working on physical health-related outcomes

Physical health-related outcomes reported in included studies were activities of daily living, long term care, frailty and physical fitness/activity. Two studies reported a positive effect and two further studies found a positive effect for some workers. One reported a neutral effect, and three further studies reported a neutral effect only for some workers. Two studies reported adverse effects for some workers (see Fig. 4).

The first of the studies finding positive effects was a cohort study with 3 year follow up of people over the age of 65 in Japan [29]. Men who continued working had less likelihood of requiring long term care (but more likelihood of decline in activities of daily living) than those who retired (OR 0.22 95% CI 0.09–0.54). Women who continued working past 65 had less likelihood both of requiring long term care (0.32 CI 0.15–0.68) and lower risk of decline in activities of daily living (0.39 95%CI 0.16–0.99) than those who retired.

An analysis of US data found fewer physical functioning difficulties in those who continued working (0.49 95% CI 0.31–0.67 per every 10 years increase in age for those in full-time work, versus 0.63 95% CI 0.54–0.72) [30]. Physical functioning difficulties amongst those both employed and retired were higher for women, those with low education, and those with low non-housing financial wealth. A positive effect for males only was reported by a cohort study from Japan [31]. Males who were working were less likely to decline in activities of daily living than those who were not working (21.2% decline versus 50% decline *p* < 0.05 in urban area, 41.6% versus 65.5% in rural area *p* < 0.01).

A study from the US reported that while there may be positive physical effects by extending working for those in average reward jobs (predicted increase in frailty of 0.31 at retirement versus increase of 0.28 if working), this was reversed for those in low reward jobs (predicted increase in frailty of 0.28 for retirees compared to 0.48 for those staying in the labour force) [32]. A study from Australia [26] similarly indicated that while there was no evidence of a significant difference in physical functioning between those who were working, and those who were voluntarily retired (*p* = 0.687), the physical health of workers in poor quality jobs (lack of control over work time, skill use or fair reward) declined more than for those who were voluntarily retired (− 4.90, 8.52−− 1.29).

Two studies provided evidence of a neutral effect of working longer on physical fitness [23] One used probability modelling and controlled for both individual status and financial position [24].

#### The effect of extended working on quality of life

We identified only one study which reported quality of life as an outcome, with differential effects for workers who were in paid employment to keep active or for enjoyment (positive effect) versus those who in employment for financial reasons (negative effect) [6]. The authors noted that caring for someone eroded the reported positive effect.

#### The effect of extended working life on other health-related outcomes

A review of 19 papers on shift work tolerance [19] concluded that there was no evidence of a negative effect of working shifts amongst older employees. Another review [20] examining injuries and accidents amongst older employees reported that sickness absence following work injury increased in each decade (median of 5 days for those aged 20–24 years versus 18 days for those aged 65 years or over).

#### Interventions

We identified only one evaluation of an intervention [33]. This initiative enabled employees to adopt flexible working options, and also provided training programmes to enable desired work/life balance. The results indicated that those who had low workability responded to the intervention in terms of maintaining their rating of ability to do their job.

A cohort study which examined a European dataset on health outcomes in retirees and older employees, reported that volunteering or engaging in sporting and social clubs had no impact on older employees (apart from those with mild depression) [34].

## Discussion

We identified 16 studies of cohort or cross-sectional design which provide evidence regarding the effects of extended working life beyond typical or statutory retirement age. One additional study evaluated an intervention. Review of this literature indicates that while there is some inconsistency, the greater weight of evidence is of positive or neutral outcomes from extended working. This evidence is particularly in regard to overall health status and physical health for most workers. Some authors have argued that in higher income countries, there has been a perception that workers should be “protected” from adverse effects of longer working lives, which has led to discouragement of extending working and entrenched employers’ attitudes regarding their ageing workforce [12]. This review adds to evidence from previous reviews that there can be health benefits from working longer [12].

While positive effects are encouraging, the evidence of neutral effects on health could also be viewed as a beneficial outcome, as it is known that working longer improves personal finances in retirement. If employment can be continued with no adverse health effects, then this could be a gain for those with limited pensions. Further analysis of these headline findings however, indicates that the benefits reported are most likely to be for males, those reducing to part time working, and those employees who are in jobs which are not low quality or low reward.

Evidence regarding the effect on mental health was more mixed than overall self-rated health, but also indicated neutral or positive effects for most workers. Other authors however, have suggested that retirement has a beneficial effect on mental health [35]. If mental health can be enhanced by retiring, then this provides an argument against extending working. The small body of evidence on quality of life effects found in our review may be suggestive of an emphasis on health for employability, with a more limited focus on understanding of work as one contributor to overall quality of life [7].

Some authors argue that any positive health effects of extending working may solely be the result of those in better health continuing in employment, whereas those in poorer health retire. However, a study in this review which adjusted for health status still found a positive effect of extended working (although the effect was of a lower magnitude) [27]. Our review suggests that health differences may not be the only explanation for the positive effects of continuing employment for some groups.

Few sources distinguished between those who had retired voluntarily by choice, and those whose decision-making was constrained by economic, health, or personal situations. Choice however, is important as those who choose to remain in employment benefit to a greater degree than those working due to financial necessity [6]. The role of health, financial and other constraints on an individual’s choice whether to continue working or retire, further highlights that while extended working may be positive for some, there may be adverse effects for others.

Differing career pathways and employment patterns for men versus women are important to consider. Women are more likely to have been employed part time during their career [25], which may preclude being able to make a choice about labour market exit due to poor pension provision. Levels of poverty are known to be higher amongst part time workers [36]. Authors have argued that the caregiving responsibilities of women should be adequately recognised and supported in order to help extend working life [37].

Many studies did not provide details of the working pattern of employees, although it can be conjectured that a sizeable number of those “extended employment” groups were not working full time, given that one study reported that of those continuing employment 45% worked less than 20 h per week [27]. Much of the evidence may therefore be comparing working part time versus retiring, which adds to the complexity in interpretation of the findings.

Of concern to potential widening of inequalities, the opportunity to reduce to part time working is more available to some employees than others [25], although it is known to enable those with a disability to extend employment [38], and be of particular benefit to those in physically demanding jobs [39].

The evidence highlights that the type of employment is important when considering health outcomes, and the potential for widening gaps in health inequalities as a result of extended working lives. Authors have described disadvantaged workers as being “virtually invisible” in policies to increase state pension age, and have starkly portrayed the struggles of workers with low levels of health and education to continue in physically demanding jobs [40]. Preferences for earlier retirement are associated with low recognition and high job demands [41], and the nature of work and health are both of key importance in retirement decision-making [42].

Attitudes to extended working are known to be influenced by not only financial gain, but also having flexible employment conditions [43]. If working lives are to be extended for those who are able to choose, employers will need to overcome workers typical preference for earlier rather than delayed retirement [44]. It has been highlighted that participation of older adults in the workforce requires consideration of both the supply side (whether employees need, want and are healthy enough to work), and the demand side (whether employees enable extended working for example by training or offering other adaptations to continue working) [12]. There has been a call for creativity in developing approaches to continued work participation [45].

The type of employment and working conditions matter in relationships between health and work [39]. The absence of studies on interventions to enable healthy extended working lives is concerning. Policies extending employment need to be accompanied by changing workplace practices to address potentially adverse effects on some population sub-groups. It has been suggested that social benefits are required to compensate for the loss of earnings and subsequent effect on income and pension for those from poorer households [46]. A cumulative disadvantage from extending employment may result, with accumulating work exposures among less advantaged population groups potentially contributing to their trajectories of greater ill health [47]. Researchers point to the “genuine protective effects” of better jobs on musculoskeletal disorders, mental health and general health [48]. Evidence from this review adds to the calls for “new tools, training methods, and organisational practices” to promote successful ageing at work [49].

### Limitations

We acknowledge the challenges in defining “typical retirement age” and our definition of extended working as employment beyond age 64 may have excluded literature with relevant findings. Many studies which we identified but rejected, recruited wide age ranges of people who were working or retired. However, we believe that a strength of this work was the specificity of evidence only to those who would be classified as extending working, rather than all older workers. We acknowledge that studies published in languages other than English were excluded, which may have resulted in relevant sources not being scrutinised.

A sizeable volume of studies were excluded from this review due to their focus on the effects of retirement, rather than outcomes from extended working. While this may seem to be a somewhat arbitrary distinction, there is a substantial body of literature available which has specifically examined the health effects of withdrawing from the labour market [35], and leaving employment for different socio-economic groups [50]. Authors have highlighted that “we know quite a lot on the implications of retirement, but less on the impact of staying in work” [5].

## Conclusions

There is evidence from cohort and cross-sectional studies of benefit or a neutral effect on overall health status and physical health from extending working. These effects are for some workers, but not all. Evidence regarding the effect on mental health was more mixed and there is little evidence regarding the effect on quality of life. The benefits reported are most likely to be for males, those reducing to part time working, and those employees who are in jobs which are not low quality or low reward. There is a clear gap in regard to interventions which have the aim of enabling older workers to extend their healthy working life, with this of particular importance for workers in employment with greater risk of adverse outcomes. We were able to identify only a single evaluation of an initiative to enhance the health of workers of typical retirement age. There is the potential for widening health inequalities between those who can choose to reduce their working hours, and those who need to continue working full time for financial reasons.

## Supplementary Information

**Additional file 1.**

## Data Availability

All data generated or analysed during this study are included in this published article and its supplementary information files.

## References

[1] Welsh J, Strazdins L, Charlesworth S, Kulik CT, D'Este C (2018). Losing the workers who need employment the most: how health and job quality affect involuntary retirement. Labour Ind.

[2] Shelton N, Head J, Carr E, Zaninotto P, Hagger-Johnson G, Murray E. Gender differences and individual, household, and workplace characteristics: Regional geographies of extended working lives. Popul Space Place. 2019;25(2):e2213. 10.1002/psp.2213. 10.1002/psp.2213 PMC7893678 33664632

[3] Edge CE, Cooper AM, Coffey M (2017). Barriers and facilitators to extended working lives in Europe: a gender focus. Public Health Rev.

[4] Modini M, Joyce S, Mykletun A, Christensen H, Bryant RA, Mitchell PB, Harvey SB (2016). The mental health benefits of employment: results of a systematic meta-review. Australas Psychiatry.

[5] van der Mark-Reeuwijk KG, Weggemans RM, Bultmann U, Burdorf A, Deeg DJH, Geuskens GA (2019). Health and prolonging working lives: an advisory report of the health Council of the Netherlands. Scand J Work Environ Health.

[6] Di Gessa G, Corna L, Price D, Glaser K (2018). The decision to work after state pension age and how it affects quality of life: evidence from a 6-year English panel study. Age Ageing.

[7] Winkelmann-Gleed A (2012). Retirement or committed to work?: Conceptualising prolonged labour market participation through organisational commitment. Empl Relat.

[8] Eisenberg-Guyot J, Peckham T, Andrea SB, Oddo V, Seixas N, Hajat A (2020). Life-course trajectories of employment quality and health in the U.S.: A multichannel sequence analysis. Soc Sci Med.

[9] De Preter H, Van Looy D, Mortelmans D (2013). Individual and institutional push and pull factors as predictors of retirement timing in Europe: a multilevel analysis. J Aging Stud.

[10] Le Blanc PM, Peeters MCW, Van der Heijden BIJM, van Zyl LE. To leave or not to leave? A multi-sample study on individual, job-related, and organizational antecedents of employability and retirement intentions. Front Psychol. 2019;10. 10.3389/fpsyg.2019.02057. 10.3389/fpsyg.2019.02057 PMC6746944 31551888

[11] Cheung F, Wu AMS (2013). Older workers' successful aging and intention to stay. J Manag Psychol.

[12] Staudinger UM, Finkelstein R, Calvo E, Sivaramakrishnan K (2016). A global view on the effects of work on health in later life. Gerontologist..

[13] Organisation for Economic Collaboration and Development (2019). Pensions at a glance 2019: OECD and G20 indicators.

[14] Critical Skills Appraisal Programme. Checklists for quality appraisal. 2009. Oxford: CASP.

[15] Baxter SK, Blank L, Woods HB, Payne N, Rimmer M, Goyder E (2014). Using logic model methods in systematic review synthesis: describing complex pathways in referral management interventions. BMC Med Res Methodol.

[16] Crowther M, Avenell A, MacLennan G, Mowatt G (2011). A further use for the harvest plot: a novel method for the presentation of data synthesis. Res Synth Methods.

[17] Ogilvie D, Fayter D, Petticrew M, Sowden A, Thomas S, Whitehead M, Worthy G (2008). The harvest plot: a method for synthesising evidence about the differential effects of interventions. BMC Med Res Methodol.

[18] Page MJ, McKenzie JE, Bossuyt PM, Boutron I, Hoffmann TC, Mulrow CD (2021). The PRISMA 2020 statement: an updated guideline for reporting systematic reviews. Br Med J.

[19] Blok MM, de Looze MP (2011). What is the evidence for less shift work tolerance in older workers?. Ergonomics..

[20] Farrow A, Reynolds F (2012). Health and safety of the older worker. Occup Med.

[21] Kajitani S (2011). Working in old age and health outcomes in Japan. Japan World Econ.

[22] Okamoto S, Okamura T, Komamura K (2018). Employment and health after retirement in Japanese men. Bull World Health Organ.

[23] Carrino L, Glaser K, Avendano M. Later pension, poorer health? Evidence from the new state pension age in the UK: University Library of Munich, Germany; 2018.

[24] Anxo D, Ericson T, Miao C (2019). Impact of late and prolonged working life on subjective health: the Swedish experience. Eur J Health Econ.

[25] McDonough P, Worts D, Corna LM, McMunn A, Sacker A (2017). Later-life employment trajectories and health. Adv Life Course Res.

[26] Welsh J, Strazdins L, Charlesworth S, Kulik CT, Butterworth P (2016). Health or harm? A cohort study of the importance of job quality in extended workforce participation by older adults. BMC Public Health.

[27] Di Gessa G, Corna LM, Platts LG, Worts D, McDonough P, Sacker A (2017). Is being in paid work beyond state pension age beneficial for health? Evidence from England using a life-course approach. J Epidemiol Community Health.

[28] Minami U, Nishi M, Fukaya T, Hasebe M, Nonaka K, Koike T, Suzuki H, Murayama Y, Uchida H, Fujiwara Y (2015). Effects of the change in working status on the health of older people in Japan. PLoS One.

[29] Tomioka K, Kurumatani N, Hosoi H (2018). Beneficial effects of working later in life on the health of community-dwelling older adults. Geriatr Gerontol Int.

[30] Stenholm S, Westerlund H, Salo P, Hyde M, Pentti J, Head J, Kivimäki M, Vahtera J (2014). Age-related trajectories of physical functioning in work and retirement: the role of sociodemographic factors, lifestyle and disease. J Epidemiol Community Health.

[31] Fujiwara Y, Shinkai S, Kobayashi E, Minami U, Suzuki H, Yoshida H, Ishizaki T, Kumagai S, Watanabe S, Furuna T, Suzuki T (2016). Engagement in paid work as a protective predictor of basic activities of daily living disability in Japanese urban and rural community-dwelling elderly residents: an 8-year prospective study. Geriatr Gerontol Int.

[32] Kalousova L, de Leon CM (2015). Increase in frailty of older workers and retirees predicted by negative psychosocial working conditions on the job. Soc Sci Med.

[33] Morelock JC, McNamara TK, James JB (2017). Workability and requests for flexible work arrangements among older adults: the role of a time and place management intervention. J Appl Gerontol.

[34] Potocnik K, Sonnentag S (2013). A longitudinal study of well-being in older workers and retirees: the role of engaging in different types of activities. J Occup Organ Psychol.

[35] van der Heide I, van Rijn RM, Robroek SJW, Burdorf A, Proper KI (2013). Is retirement good for your health? A systematic review of longitudinal studies. BMC Public Health.

[36] Horemans J, Marx I, Nolan B (2016). Hanging in, but only just: part-time employment and in-work poverty throughout the crisis. J Eur Labor Stud.

[37] Carr E, Murray ET, Zaninotto P, Cadar D, Head J, Stansfeld S (2018). The association between informal caregiving and exit from employment among older workers: prospective findings from the UK household longitudinal study. J Gerontol B Psychol Sci Soc Sci.

[38] Pagan R (2012). Transitions to part-time work at older ages: the case of people with disabilities in Europe. Disabil Soc.

[39] Hess M, Bauknecht J, Pink S (2018). Working hours flexibility and timing of retirement: findings from Europe. J Aging Soc Policy.

[40] Lain D, Phillipson C (2019). Extended work lives and the rediscovery of the 'Disadvantaged' older worker. Generations J Am Soc Aging.

[41] Carr E, Hagger-Johnson G, Head J, Shelton N, Stafford M, Stansfeld S, Zaninotto P (2016). Working conditions as predictors of retirement intentions and exit from paid employment: a 10-year follow-up of the English longitudinal study of ageing. Eur J Ageing.

[42] de Wind A, Scharn M, Geuskens GA, van der Beek AJ, Boot CRL (2018). Predictors of working beyond retirement in older workers with and without a chronic disease - results from data linkage of Dutch questionnaire and registry data. BMC Public Health.

[43] Carlstedt AB, Brushammar G, Bjursell C, Nystedt P, Nilsson G (2018). A scoping review of the incentives for a prolonged work life after pensionable age and the importance of "bridge employment". Work.

[44] Hess M (2018). Expected and preferred retirement age in Germany. Z Gerontol Geriatr.

[45] Steenstra I, Cullen K, Irvin E, Van Eerd D (2017). Team IWHOWR. A systematic review of interventions to promote work participation in older workers. J Saf Res.

[46] Wels J (2019). Assessing the association between late career working time reduction and retirement plans. A cross-National Comparison Using the 2012 labour force survey ad hoc module. Soc Policy Soc.

[47] Lahelma E, Pietilainen O, Chandola T, Hyde M, Rahkonen O, Lallukka T. Occupational social class trajectories in physical functioning among employed women from midlife to retirement. BMC Public Health. 2019;19:1525. 10.1186/s12889-019-7880-0. 10.1186/s12889-019-7880-0 PMC6857143 31727156

[48] Henseke G (2018). Good jobs, good pay, better health? The effects of job quality on health among older European workers. Eur J Health Econ.

[49] Ackerman PL, Kanfer R (2020). Work in the 21st century: new directions for aging and adult development. Am Psychol.

[50] Schaap R, de Wind A, Coenen P, Proper K, Boot C (2018). The effects of exit from work on health across different socioeconomic groups: a systematic literature review. Soc Sci Med.

