# Supplementary material for: Is working in later life good for your health? A systematic review of health outcomes resulting from extended working lives
Source: BMC Public Health. 2021 Jul 9;21:1356. doi: 10.1186/s12889-021-11423-2 (PMC8268509; doi:10.1186/s12889-021-11423-2)
Supplement: Supplementary file 1 — Additional file 1. [file 12889_2021_11423_MOESM1_ESM.docx]

**Supplementary file**

**Is working in later life good for your health? A systematic review of health outcomes resulting from extended working lives**

*Susan Baxter, School for Health and Related Research, University of Sheffield, UK

Lindsay Blank, School for Health and Related Research, University of Sheffield, UK

Anna Cantrell, School for Health and Related Research, University of Sheffield, UK

Elizabeth Goyder, School for Health and Related Research, University of Sheffield, UK

*Corresponding author

Dr Susan Baxter, School for Health and Related Research, University of Sheffield, Regent Court, Regent Street, Sheffield, S14DA

Email: [s.k.baxter@sheffield.ac.uk](mailto:s.k.baxter@sheffield.ac.uk)

**Search strategy**

Database: Ovid MEDLINE(R) and Epub Ahead of Print, In-Process & Other Non-Indexed Citations and Daily <1946 to February 11, 2020>

Search Strategy:

--------------------------------------------------------------------------------

1 older work$.ab,ti. (1332)

2 elderly work$.ab,ti. (104)

3 late employment.kw. (1)

4 (older workers or older working population).kw. (127)

5 elderly workers.kw. (5)

6 "extending working life".ab,ti. (13)

7 "Extending working life".kw. (5)

8 1 or 2 or 3 or 4 or 5 or 6 or 7 (1483)

9 health.ab,ti. (1786973)

10 (wellbeing or well being or wellness).ab,ti. (94017)

11 health status/ or health status disparities/ (93684)

12 inequalit$.ab,ti. (28791)

13 economic$ outcome$.ab,ti. (3061)

14 societ$ outcomes.ab,ti. (121)

15 community outcome$.ab,ti. (144)

16 "Community functioning".kw. (18)

17 9 or 10 or 11 or 12 or 13 or 14 or 15 or 16 (1864677)

18 8 and 17 (703)

19 limit 18 to (english language and humans and yr="2011 -Current") (284)

**2. Websites searched for grey literature**

**Department for Work and Pensions** <https://www.gov.uk/government/organisations/department-for-work-pensions>

A new vision for older workers: retain retrain recruit <https://www.gov.uk/government/publications/a-new-vision-for-older-workers-retain-retrain-recruit>

Fuller working lives a partnership approach <https://www.gov.uk/government/publications/fuller-working-lives-a-partnership-approach>

Fuller working lives evidence base 2017 <https://www.gov.uk/government/publications/fuller-working-lives-evidence-base-2017>

Fuller working lives: a framework for action <https://www.gov.uk/government/publications/fuller-working-lives-a-framework-for-action>

Attitudes to working in later life British social attitudes 2015 <https://www.gov.uk/government/publications/attitudes-to-working-in-later-life-british-social-attitudes-2015>

Older workers and the workplace evidence from the workplace employment relations survey <https://www.gov.uk/government/publications/older-workers-and-the-workplace-evidence-from-the-workplace-employment-relations-survey>

Age Action Alliance and DWP Employer Toolkit <https://assets.publishing.service.gov.uk/government/uploads/system/uploads/attachment_data/file/411428/older-workers-employer-toolkit-summary.pdf>

Employer experiences of recruiting retaining and retraining older workers qualitative research <https://www.gov.uk/government/publications/employer-experiences-of-recruiting-retaining-and-retraining-older-workers-qualitative-research>

Management of an ageing workforce <https://www.gov.uk/government/publications/management-of-an-ageing-workforce>

Future of ageing seminar on older workers <https://www.gov.uk/government/publications/future-of-ageing-seminar-on-older-workers>

Future of ageing workplace infrastructure <https://www.gov.uk/government/publications/future-of-ageing-workplace-infrastructure>

Future of ageing changing work requirements and environments <https://www.gov.uk/government/publications/future-of-ageing-changing-work-requirements-and-environments>

Sector based work academics and work experience trials for older claimants combined quantitative and qualitative findings <https://www.gov.uk/government/publications/sector-based-work-academies-and-work-experience-trials-for-older-claimants-combined-quantitative-and-qualitative-findings>

Extending working life sector initiative <https://www.gov.uk/government/publications/extending-working-life-sector-initiative>

Attitudes to age in Britain in house research no 7 <https://www.gov.uk/government/publications/attitudes-to-age-in-britain-201011-in-house-research-no-7>

Default retirement age employer qualitative research <https://www.gov.uk/government/publications/default-retirement-age-employer-qualitative-research-rr672>

Review of the default retirement age summary of the stakeholder evidence <https://www.gov.uk/government/publications/review-of-the-default-retirement-age-summary-of-the-stakeholder-evidence-rr675>

2010 to 2015 government policy: older people <https://www.gov.uk/government/publications/2010-to-2015-government-policy-older-people>

**Centre for Ageing Better** <https://www.ageing-better.org.uk/>

The State of Ageing in 2019: adding life to our years <https://www.ageing-better.org.uk/publications/state-of-ageing-2019>

Supporting carers back into work: insights form the working potential project <https://www.ageing-better.org.uk/publications/supporting-carers-back-to-work-insights-working-potential-project>

Priorities for government: transforming later lives <https://www.ageing-better.org.uk/sites/default/files/2019-10/Priorities-for-government-Transforming-later-lives.pdf>

Mid-life support: insights for employers <https://www.ageing-better.org.uk/publications/mid-life-support-insights-employers>

Employment support for over 50s: rapid evidence review <https://www.ageing-better.org.uk/publications/employment-support-over-50s-evidence-review>

The experience of the transition to retirement: rapid evidence review <https://www.ageing-better.org.uk/publications/transition-to-retirement-rapid-evidence-review>

Thinking ahead: exploring support provided by employers to help staff plan for their future <https://www.ageing-better.org.uk/publications/thinking-ahead-exploring-support>

Developing the mid-life MOT <https://www.ageing-better.org.uk/publications/developing-mid-life-mot>

Becoming an age-friendly employer <https://www.ageing-better.org.uk/publications/becoming-age-friendly-employer>

Health warning for employers: supporting older workers with health conditions <https://www.ageing-better.org.uk/publications/health-warning-employers>

A silver lining for the UK economy? The intergenerational case for supporting longer working lives <https://www.ageing-better.org.uk/publications/silver-lining-uk-economy>

Inequalities in later life <https://www.ageing-better.org.uk/publications/inequalities-later-life>

Inequalities in later life: the issue and the implications for policy and practice <https://www.ageing-better.org.uk/publications/inequalities-later-life-issue-and-implications-policy-and-practice>

Addressing worklessness and job insecurity amongst people aged 50 and over in Greater Manchester <https://www.ageing-better.org.uk/publications/addressing-worklessness-and-job-insecurity-amongst-people-aged-50-and-over-greater>

Fulfilling work: what do older workers value about work and why? <https://www.ageing-better.org.uk/publications/fulfilling-work-what-do-older-workers-value-about-work-and-why>

Later life in 2015: an analysis of the views and experiences of people aged 50 years and over <https://www.ageing-better.org.uk/publications/later-life-2015-analysis-views-and-experiences-people-aged-50-and-over>

**Age UK** [www.ageuk.org.uk](http://www.ageuk.org.uk)

Behind the headlines: why the employment rate does not tell the whole story about working longer <https://www.ageuk.org.uk/globalassets/age-uk/documents/reports-and-publications/reports-and-briefings/active-communities/rb_april17_behind_the_headlines_-hours_worked.pdf>

Walking the tightrope the challenges of combining work and care in later life <https://www.ageuk.org.uk/globalassets/age-uk/documents/reports-and-publications/reports-and-briefings/active-communities/rb_july16_walking_the_tightrope.pdf>

A means to many end older workers’ experiences of flexible working <https://www.ageuk.org.uk/globalassets/age-uk/documents/reports-and-publications/reports-and-briefings/active-communities/rb_sept12_a_means_to_many_ends_older_workers_experiences_of_flexible_working.pdf>

**The Centre for research into the older workforce** <https://www.agediversity.org/>

What are the supply (workforce) and demand (product) implications of an ageing society <https://www.agediversity.org/wp-content/uploads/2019/03/Foresight-future-ageing-workforces-manufacturing.pdf>

Wellbeing, Health, Retirement and the Lifecourse <https://wherl.ac.uk/about/findings/> finding from the 3 year research project

The Progressive Policy Think Tank. 2017. Extending working lives Report <https://www.ippr.org/publications/extending-working-lives>

**University of Kent Extending Working lives** <https://www.kent.ac.uk/extendingworkinglives/>

The International Longevity Centre UK. 2013. Extending working lives: a provocation <https://ilcuk.org.uk/wp-content/uploads/2018/10/Extending-working-lives-a-provocation.pdf>

MRC Lifelong Health and Wellbeing extending working lives awards <https://mrc.ukri.org/documents/pdf/extending-working-lives-awards/>

Eurofund. 2017. Extending working lives: what do workers want? <https://www.eurofound.europa.eu/publications/report/2017/eu-member-states/extending-working-life-what-do-workers-want>

**NHS**

Working Longer Review. 2014. Preliminary findings and recommendations report for the health departments <https://www.nhsemployers.org/-/media/Employers/Documents/Pay-and-reward/WLR-Preliminary-findings-and-recommendations-report.pdf?la=en&hash=6DEF00660C4B5B612E671163A500B603CA20F431>

**University College London**. renEWL Publications. <https://www.ucl.ac.uk/epidemiology-health-care/research/epidemiology-and-public-health/research/renewl/publications>

**UK Research and Innovation**. EXTEND: Social inequalities in extending working lives of an ageing workforce Publications. <https://gtr.ukri.org/projects?ref=ES%2FP000177%2F1>

**Institute for employment studies** (ies) <https://www.employment-studies.co.uk/publications>

Managing extended working life <https://www.employment-studies.co.uk/resource/managing-extended-working-life>

**3. Studies excluded at full paper scrutiny**

1. Ackerman PL, Kanfer R. Work in the 21st century: New directions for aging and adult development. American Psychologist. 2020;75(4):486-98.

2. Ardito C LR, Blane D, D’Errico A,. To work or not to work? The effect of higher pension age on cardiovascular health. Turin, Italy: Laboratorio Revelli; 2016.

3. Bratberg E, Holmås TH, Monstad K. Health effects of reduced workload for older employees. Health Econ. 2020;29(5):554-66.

4. Carlstedt AB, Brushammar G, Bjursell C, Nystedt P, Nilsson G. A scoping review of the incentives for a prolonged work life after pensionable age and the importance of "bridge employment". Work-a Journal of Prevention Assessment & Rehabilitation. 2018;60(2):175-89.

5. Carmichael F, Duberley J, Szmigin I. Older women and their participation in exercise and leisure-time physical activity: the double edged sword of work. Sport in Society. 2015;18(1):42-60.

6. Carmichael F, Hulme C, Porcellato L. Older age and ill-health: links to work and worklessness. International Journal of Workplace Health Management. 2013;6(1):54-65.

7. Cho E, Chen T-Y. The Effects of Work–Family Experiences on Health Among Older Workers. Psychology and Aging. 2018;33(7):993.

8. Cottini E, Ghinetti P. Health Effects of Risky Lifestyles and Adverse Working Conditions: Are Older Individuals More Penalized? British Journal of Industrial Relations. 2019.

9. Davies R, Jones M, Lloyd-Williams H. Age and Work-Related Health: Insights from the UK Labour Force Survey. British Journal of Industrial Relations. 2016;54(1):136-59.

10. Demou E, Bhaskar A, Xu T, Mackay DF, Hunt K. Health, lifestyle and employment beyond state-pension age. BMC Public Health. 2017;17.

11. Flower DJC, Tipton MJ, Milligan GS. Considerations for physical employment standards in the aging workforce. Work. 2019;63(4):509-19.

12. Gommans FG, Jansen NW, Mackey MG, Stynen D, de Grip A, Kant IJ. The Impact of Physical Work Demands on Need for Recovery, Employment Status, Retirement Intentions, and Ability to Extend Working Careers: A Longitudinal Study Among Older Workers. J Occup Environ Med. 2016;58(4):e140-51.

13. Harber-Aschan L, Chen WH, McAllister A, Koitzsch Jensen N, Thielen K, Andersen I, et al. The impact of longstanding illness and common mental disorder on competing employment exits routes in older working age: A longitudinal data-linkage study in Sweden. PLoS ONE. 2020;15(2):e0229221.

14. Ide K, Tsuji T, Kanamori S, Jeong S, Nagamine Y, Kondo K. Social Participation and Functional Decline: A Comparative Study of Rural and Urban Older People, Using Japan Gerontological Evaluation Study Longitudinal Data. International Journal of Environmental Research and Public Health. 2020;17(2).

15. Jones C, Routley V, Trytell G, Ibrahim J, Ozanne-Smith J. A descriptive analysis of work-related fatal injury in older workers in Australia 2000-2009. Int J Inj Contr Saf Promot. 2013;20(1):85-90.

16. Jones MK, Latreille PL, Sloane PJ, Staneva AV. Work-related health risks in Europe: are older workers more vulnerable? Soc Sci Med. 2013;88:18-29.

17. Lain D, Phillipson C. Extended Work Lives and the Rediscovery of the 'Disadvantaged' Older Worker. Generations-Journal of the American Society on Aging. 2019;43(3):71-7.

18. Nahum-Shani I, Bamberger PA. Work hours, retirement, and supportive relations among older adults. Journal of Organizational Behavior. 2011;32(2):345-69.

19. Nilsson K. Interventions to reduce injuries among older workers in agriculture: A review of evaluated intervention projects. Work. 2016;55(2):471-80.

20. Nyqvist F, Forsman AK, Cattan M. A comparison of older workers' and retired older people's social capital and sense of mastery. Scand J Public Health. 2013;41(8):792-8.

21. Peng L, Chan AHS. A meta-analysis of the relationship between ageing and occupational safety and health. Safety Science. 2019;112:162-72.

22. Poscia A, Moscato U, La Milia DI, Milovanovic S, Stojanovic J, Borghini A, et al. Workplace health promotion for older workers: a systematic literature review. BMC Health Serv Res. 2016;16 Suppl 5:329.

23. Rodrigues FR, Pina e Cunha M, Castanheira F, Bal P, Jansen PG. Person-job fit across the work lifespan-The case of classical ballet dancers. Journal of Vocational Behavior Vol 118 2020, ArtID 103400. 2020;118.

24. Sewdas R, Thorsen SV, Boot CRL, Bjorner JB, Van der Beek AJ. Determinants of voluntary early retirement for older workers with and without chronic diseases: A Danish prospective study. Scand J Public Health. 2020;48(2):190-9.

25. Soderbacka T, Nyholm L, Fagerstrom L. Workplace interventions that support older employees' health and work ability-a scoping review. BMC Health Serv Res. 2020;20(1).

26. Staudinger UM, Finkelstein R, Calvo E, Sivaramakrishnan K. A Global View on the Effects of Work on Health in Later Life. Gerontologist. 2016;56 Suppl 2:S281-92.

27. van Zon SK, Bultmann U, Reijneveld SA, de Leon CF. Functional health decline before and after retirement: A longitudinal analysis of the Health and Retirement Study. Soc Sci Med. 2016;170:26-34.

**4. Extraction table**

| **Author**  **Year**  **Location**  **Health outcomes /Intervention** | **Population** | **Study design** | **Outcomes** | **Main findings** | **Conclusion and**  **other notes** |
| --- | --- | --- | --- | --- | --- |
| Anxo 2019  Sweden | Age 65+ retirees  Survey n= 8022  retired individuals. 34% retired before 65, 41% retired at 65 and 25% retired after 65. | Longitudinal routine data (Longitudinal Integration Database for Health Insurance and Labour Market Studies) plus a postal survey sent to 20,000. Probability model (standard linear model adjusted for SES). Compared retired vs. stayed in workplace @65  Total sick days used to proxy health before age 65.  Treatment group defined as worked at least half a year after 65 | Self-assessed health via postal survey, 5 point Likert scale very bad to excellent.  Also self-reported physical fitness, self-reported depressive symptoms, well-being. | 6.8% higher probability in reporting better health in retirement for those who continued to work after 65 compared to those retired at 65. But advantage estimated would be lost after 6 years. Mean health difference between the two groups 0.213 p<0.01.  Linear probability model estimated treatment effect 0.139, suggesting that having work experience after the retirement age of 65 would on average increase the probability of reporting a better health during retirement by about 14%. This figure adjusted to 0.109 controlled for demographics and socio-economic characteristics. The prolonging working life after age of 65 and better health association remains even after controlling for pre-retirement health condition. Estimated effect 0.068 p<0.01, those delaying retirement had about 6.8% probability of reporting better health in retirement.  No statistically significant effect of working longer on other outcomes physical fitness, self-reported depressive symptoms or well-being (life satisfaction).  Those working longer had average higher educational attainment, higher labour and capital income, fewer unemployment days (average 6.7 days over 5 years), and took average 6 fewer days sickness per year.  The positive health effect of working after 65 is restricted to male respondents, married people, and medium skilled workers. Not statistically significant for females, single, or low/high skilled (although small number of observations for these two skill groups). | Explores the relationship between the prolongation of working life and subjective health.  Better self-reported overall health in retirement for those who work beyond 65 (males, married, middle skilled), a weak and transitory but positive effect.  Healthier people more likely to delay retirement. |
| Ardito 2016  Italy | Male retired (defined as receiving an occupational pension) and those still in employment after pension age.  Males employed and self-employed workers in private sector.  Aged 68-70.  (N=94,52). | Retrospective analysis of routine data: WHIP&Health database – social security and hospital discharge data. Study used a random 7% sample. | Cardio vascular disease incidence | Retirees who, during their careers, were lower income earners, and manual workers are likely to experience the impact of pension age on CVD to a greater extent (increase of 4.4%, p<0.01)  A one-year delay in retirement increases the incidence of hospitalization for cardiovascular diseases (CVD) at 68-70 years old by 2.4 percentage points (p-value<0.01). | Results show a significant health detrimental effect of extended working life.  Retirees exposed to more disadvantageous  working conditions face higher CVD risks as a result of retiring at older age.  Effects of pension reform and seasonal working in Italy on retirement age noted.  Note: mean pension age in group was 59 years. |
| Blok 2011  Netherlands | Older than 64 | Review  19 papers  Shift work tolerance in older workers.  English language.  Two intervention studies all others associational studies, various sectors of industries. | Age-shift work interaction effect on sleep, fatigue, performance, accidents and health. Studies on age–shift (morning, afternoon, night) and age–shift system (roster) | Did not find evidence overall for the suggestion of more shift work problems in older workers.  Two studies reported more problems in older people, four studies reported opposite results, while in ﬁve studies no signiﬁcant age–shift work interaction was observed. Older compared with younger workers have more sleep problems with night shifts, while the opposite is true for morning shifts.  Many papers reported high inter-individual differences in tolerance. | Effects of shift work not increased overall with older workers. |
| Carmichael 2013  UK | 50-68 years  N=56 (M+F)  North West England, recruited via community venues and groups. Just over half male, most married or co-habiting, 30% retired, 39% in paid employment. Median household income £20,000-£30,000. | Qualitative interviews and some quantitative analysis (British Household Panel Survey) | Physical/mental health  Labour market participation  Both relationships | Two-way causality in relationships between health, work and worklessness in older age.  Type of job and workplace conditions matter.  Impact of ill health on work participation accentuated by age for women only.  The impact of age on health can be detrimental to employment, potentially limiting the type of job or work an older worker can do, how well they can do it and possibly influencing employers’ attitude to their employability. | Relationship between health and work is two way. Pension age rises should be accompanied by interventions to improve health and change workplace practices to facilitate longer working lives. |
| Carrino 2018  Italy (Data from UK) | Women aged 60-64 average age 62.5, 70% had a partner, 80% at least GCSE. 40% manual-routine work, 30% intermediate, 30% higher occupations. Excluded those who had never worked.  N=3452 | Retrospective analysis of survey data (Understanding Society) comparing women who were eligible for pensions at age 60-62 with those who had to work longer before eligibility for pension. Linear probability models. | General Health Questionnaire depression score  SF-12 mental and physical health (self-reported, Likert scale).  Self-reported employment status – paid worker, unemployed, retired, looking after family or home, long term sick/disabled | Increasing state pension age estimated to have increased employment rates by 11%, 41% of women reported being in employment, 2.8% in sickness, 3.2% in caring for family/home, and 1% in unemployment. Being below the SPA as a result of the reform leads to a reduction of £220 in monthly individual income, generated by a decrease in pension income which is not compensated by earnings from increased labour supply.  The impact of reform varies by type of employment, with those in routine manual occupations more negatively affected than those in professional occupations. There was no statistically significant impact of the reform on any outcome for intermediate and managerial categories.  The probability of alternative pathways to retirement (through sickness, home/family-caring and unemployment) were larger for women in routine-class occupations than for women in managerial occupations.  A one-month increase in the SPA increases the probability of depression by 0.2 points for women from routine occupations, with an elasticity of 0.7%. The effect is not significant for women in the intermediate and managerial categories.  The probability of alternative pathways to retirement (through sickness, home/family-caring and unemployment) are larger for women in routine-class occupations than for women in managerial occupations.  Longer postponement of SPA leads to worse depression scores for those affected compared to those not affected (GHQ increases by 0.57 points for those with an increase of 6-24 months (elasticity 5%), 0.81 for those with an increase of 24-36 months (+7.3%), and 1.19 for those with an increase of 36 or more months (+10.7%)).  Longer extensions of SPA also lead to larger reductions in the index of mental well-being. The largest effects are found for women with a postponement of three or more years (cohort 1953-1955, with an average SPA increase of 55 months): a GHQ elasticity of +10.7%, and a MCS elasticity of -3.7%.  The reform has no clear consistent impact on intermediate and managerial classes, with the effect being largely confined to women from routine occupations. The increase in SPA led to a divergence in health between occupational groups.  The rate of women reporting being sick, disabled or caring for family/home increases for cohorts who are affected by the increased state pension age. | Women in routine occupations who had a delayed statutory pension age as a result of reform had significantly higher GHQ depression scores and a decline in the SF-12 mental well-being score. Longer postponement of state pension age leads to worse mental wellbeing and depression scores for women from routine occupations (not intermediate or managerial). |
| Di Gessa 2017  UK | Men 65-74, women 60-69 in 2006/7  N=2039 analysed (n=1608 data collected at all time points)  Significant difference at p<0.05 or 0.01 between those not working versus working at baseline–worse depression, reduced somatic health, not having education, not highest wealth (men only), care provided (women only) | Retrospective analysis of survey data (English Longitudinal Study of Ageing survey of individuals in private households aged over 50) 2004/5 2006/7, 2008/9. Life history interviews for a sample.  Logistic regression. | Depression, measured by self-reported depressive symptoms in week prior.  Somatic health (combination of self-reported health and presence of one or more limitations with activities of daily living, long standing illness, self-report of doctor diagnosed heart disease/stroke, mobility limitations)  Sleep disturbance self-reported in month prior.  Nurse measured grip strength  Employment histories. | About 25% of women and 15% of men worked beyond state pension age. Around one third of those who worked beyond SPA were in managerial positions, 45% worked less than 20 hours per week and a third of men and 41% of women had sedentary jobs.  Initial analyses suggested that men and women in paid work were more likely to report better health at follow-up. Respondents in paid work beyond SPA were between 0.44 (men) and 0.57 (women) times less likely to be depressed, and between 0.64 (men) and 0.73 (women) times less likely to report sleep disturbance. They were also significantly more likely to report better somatic health (β=0.323 for men, and β=0.292 for women). However, once baseline socioeconomic characteristics as well as adulthood and baseline health and labour market histories were accounted for, the health benefits of working beyond SPA were no longer significant.  Fully adjusted results also suggest that paid work beyond SPA does not have differential effects on health depending on characteristics of the job such as physical demand, hours worked or social class. | The results suggest that being in paid work beyond SPA is not associated with better (or detrimental) health.  Those who report good health and are more socioeconomically advantaged are more likely to be working beyond SPA to begin with.  Only a select group of healthy older adults works beyond SPA. |
| Di Gessa 2018  UK | Men 65-74, women 60-69 at baseline. 56% female, 445 male, 74% some education, 24% in highest wealth quartile, 33% managerial and professionals, 40% routine and managerial, 13% carer, 73% married.  Groups of those working for financial reasons, those working voluntarily and those normal retirement, involuntary retirement or voluntary retirement. Excluded those who had never worked. N=2502 (longitudinal analysis) 1823 (cross sectional analysis) Only n=19 were still in paid work at follow up survey. | Retrospective analysis of survey data (English Longitudinal Study of Ageing, surveys individuals over 50 years) 2008/9 and 2014/15 | CASP-19 self-completion survey - dimensions of control, autonomy, self-realisation and pleasure. 0-57 scoring.  Employment status and reasons.  Activities of daily living  Long standing illness  Depression  Social relationships and contacts | Around 20% were in paid employment beyond state pension age, with two thirds of these in paid work voluntarily (13% of total sample) giving the reason that they either enjoyed working or were working to keep active and fit, the other third (7% of total sample) reported being in employment for financial reasons.  Respondents who were in paid work voluntarily (13%) reported the highest QoL (CASP-19 = 45.4) whereas the lowest QoL was reported by those who retired involuntarily (24%) (CASP-19 = 38.9).  On average respondents experienced a decrease in QoL: about a quarter experienced a decrease of 5 points or more whereas just over 16% experienced an improvement of 5 or more points. Respondents who transitioned from employment ‘for financial reasons’ to retirement were the only subgroup who experienced an increase in their CASP-19 scores.  Being in paid work out of financial necessity was significantly associated with worse QoL compared to being retired at the expected/usual age (significant only when scores were adjusted for health status (β = −1.21).  Those who continue to work for positive reasons (about two-thirds of workers) report the highest levels of QoL, (β = 1.62). These workers also experience marginal improvements in QoL when they eventually leave the labour market. Among retirees, those who reported voluntary retirement were significantly more likely to report higher QoL (β = 1.12). Caring for someone significantly reduced QoL.  In contrast, those who continue working beyond SPA out of financial necessity (one third of workers) report a CASP-19 score of about 4 points lower at baseline, and this level does not rebound upon eventual retirement.  Those involuntary retired have the poorest health profile (27% were depressed; 38% had functional limitations; 52% reported limiting LSI) whereas those in work voluntarily tended to report better health (13% depressed; 15% with limiting LSI; 8% had functional limitation), with those retired at the normal age and in work for financial reasons somewhere in between. | Paid work beyond SPA out of economic necessity is associated with lower quality of life (average 4 points lower CASP-19 scores). |
| Farrow 2012  UK | 60+ | Systematic Review  36 studies – only 6 papers included participants who were working beyond age 65 | Injuries and accidents  Sickness absence | One study reported that sickness absence following work injury increased in each decade, with a median of 5 days for those aged 20–24 years and 18 days for those aged 65 years or over. Workers over 65 were at particular risk from injuries associated with transportation/driving and had more disabling fracture injuries compared with younger workers. Reported injuries were not limited to ‘dangerous’ jobs, driving or heavy manual work.  A US survey of occupational fatalities reported that workers aged 65 or over had 6,471 fatalities (13.7 per 100,000 workers), a rate almost three times that of workers below 65 (5.1 per 100,000). This was echoed in another US survey which reported three times the risk of of fatalities in over 65s. The main causes of death for workers in the over 65 age group were machinery (28%), motor vehicles (19%), falls (13%) and homicide (13%). Machinery-related fatalities for males aged 65 or over were almost six times greater than the rate for pre-retirement age males. The fatality rate for falls amongst females aged 65 years and over was 14 times that of females aged 16–64 years.  Other studies predominantly compared people in their 60s with younger workers rather than those post statutory retirement age.  Those employed over the age of 60 may be a self-selected se the healthy worker. Relatively little is known about people who work beyond the age of 60. | There is almost no explicit research data with analysis of workers over age 65 and workplace factors associated with injury or accident. |
| Fujiwara 2016  Japan | 306 (urban Tokyo suburb) and 675 (rural village area)  Aged 65–84 years  N=981 411 males 570 females  In Tokyo 65% employees, in rural area 68% men employees, 73% women termed family workers. Rural area mostly agriculture, forestry and fishery, in Tokyo most administrative, managerial or professional. | 8-year longitudinal study | Activities of daily living (ADL) via self-reported TMIG Index of Competence, smoking status, exercise habits, Life Satisfaction Index, usual walking speed and serum albumin  Working status and frequency  Medical history | 78.8% of men and 70.4% of women were working in the urban area compared with 58.3% men and 57.0% women in the rural area.  In both areas, participants who were not working were more likely to decline in ADL than those working (P < 0.05), except for women in the urban area.  Male participants who did not engage in paid work had a higher adjusted hazard ratio of onset of BADL disability, compared with those working, but this was not seen for female participants.  Both male and female participants in the rural area had a higher risk of onset of ADL disability than the urban area. But this was no longer significant after being adjusted for usual walking speed and serum albumin (those in rural areas had lower). | Working might be protective from a decline in BADL but only for men. |
| Kajitani 2011  Japan | Age 60+  Male  N=2032  58.5% aged 60-69 and 33.4% aged 70-79. | Estimation model  Routine data  National Surveys of the Japanese Elderly 1990-1996 | Self-assessed health status  Physical limitations  Diseases, Nutrition, Mental status, Life expectancy | Around 40% of those aged 60 and over employed, just under half of these are self-employed. Pension age in Japan was 60 during 1990-1993.  Working older men tend to be healthier than those not working (Pearson test 76.34 p<0.01)  The elderly whose longest-held occupation was professional tend to be healthier than those in other industries (p<0.01). 15–25% of the elderly whose longest-held job was sales, work in agriculture, the forestry and fisheries industry, or a blue-collar job self-rated their health as ‘‘Not very good’’ or ‘‘Poor.’’  The people whose longest-held job was managerial, sales, or manual work would experience a greater deterioration of health as they age,  Japanese men prefer to work for fewer hours as they grow older. A relative decrease in labour hours, are related to the fact that the working Japanese elderly tend to be healthier. | The elderly maintain their health by working, but with a relative decrease in working hours. |
| Kalousova 2015  European + US data | Older workers  N=2475  Mean age 55 at baseline, employed or self-employed, 58% male, 43% retired between baseline and follow up, 40% high effort employment, 28% low reward, 29% low education, 33% high. | Cohort study 2004 and 20011, Survey of Health Ageing and Retirement in Europe, included US Health and Retirement Survey and English Longitudinal Study of Ageing  Multi-level regression modelling | Frailty measured by self-report and handgrip at interview, walking speed | For those in an average reward job at baseline there is a predicted increase in frailty of 0.31 at retirement. The predicted increase in frailty would be smaller for this person if he had not retired of 0.28.  For those in low reward employment at baseline there is a predicted increase in frailty of 0.28 for retirees and 0.48 for those who stayed in the labour force.  For individuals who have jobs with little reward, retirement may help with tempering their consequences for physical health. | Low reward had the most detrimental consequences for health when a respondent did not retire.  Persons in average or high reward positions did not benefit from retirement. |
| McDonough 2017  UK authors using US data | Aged 52-69 and early 70s (US retirement age 62)  N=6522 | Cohort study, data from Health and Retirement Study (1992-2012) Americans aged 50+  Logistic regression  Life course approach | Self-rated health (5 point scale poor, fair good, very good, excellent)  Functional limitations | Only around 14% of men and 10% of women worked full time until aged 65 then retired. Around 24% of women and 38% of men extended working (full time or part time). Only 2% of men worked part time early in careers. Women tended to have different career trajectories with around 13% working part time and 12% not in employment during all the years analysed. Those not in employment throughout had highest percentage reporting poor health.  Across both sexes those reducing to part-time work around age 62 or 65 were less likely than almost all other groups (except women working part time throughout) to report poor health in their early 70s (men OR 0.49 p<0.01; women OR 0.29 p<0.001)  Predicted probability of poor health for men reducing to part time at 65 =0.099 95% CI 0.058-0.139. For those working full time then retiring at 65 it was 0.220 CI 0.178-0.2623). For men working full time throughout it was 0.186 CI 0.151-0.221.  For women reducing to part time predicted probability of poor health was 0.139 CI 0.081-0.196 (reduced aged 65) and 0.133 CI 0.080-0.185 (reduced aged 62). For those working full time then retiring at 65 it was 0.228 CI 0.181-0.275.  For women all employment pathways were associated with reduced risk of poorer health than non-employment. Women had the best health if they remained employed part-time throughout (OR 0.21 p<0.000 predicted probability of poor health 0.099 95% CI 0.040-0.159) Next least likely were full time reduced to part time aged 62 or 65 (OR 0.29 p<0.000). Fourth least likely were those working full time throughout (OR 0.32 p<0.000)  Men who reduced to part time around age 65 are among the most advantaged. They are more likely to be white, college-educated and in better health in their late 40s/early 50s and early 60s; and have greater household wealth and income than most other groups.  Men and women who continued to work full time tended to be less educated and be from an ethnic minority group and have lower wealth than those who were part time or reduced to part time.  Poor health may have led to early exit around aged 57 or ongoing non-employment throughout 50’s and 60’s.  Note: overlapping 95% confidence intervals across some of the groups preclude definitive statements.  A range of marital, parental and work histories over the prime adult years had no impact on the employment-health relationship (results not shown). | Those reducing from full time to part time working at age 65 have less risk of poor health in 70s than those retiring from full time work.  There is a need for flexible employment policies that foster opportunities to work part-time. |
| Minami 2015  Japan | Age 65+ (mean 73.4) n=1768  Full-time worker (n=220), part-time worker (n=273), and non-worker (n=1275)  Population of a city near Tokyo, over half of workers commute to Tokyo for employment, 11% agricultural lands around the city. Excluded those in care homes or needing basic care. 42% male, 25% college degree or above, 16% self-employed, 64% in lowest income bracket. | Cohort study, mailed survey at three points 2008, 2010, 2012  ANCOVA analysis | Self-rated health,  Mental health (Geriatric Depression Scale 15),  Higher-Level Functional Capacity Tokyo Metropolitan Institute of Gerontology Index of Competence (activities of daily living) | Full-time and part-time workers were almost at a same level but significantly better than non-workers at self-rated health, GDS15, and TMIG-IC (p<0.001)  Estimated from chart – full time workers self-rated health 0.9, part time 0.85, non-workers 0.7. Very wide confidence interval for non-workers.  Estimated from chart - full time workers depression 4, part time 4, non-workers 5. Wide confidence interval for non-workers.  Estimated from chart functional capacity – full time 12, part time 12, non-workers 11. Wide confidence intervals.  Both mental health and HLFC in people aged 65 years and over significantly worse in those retired; especially, mental health worsened rapidly and HLFC gradually. However, these indicators didn’t worsen in subjects who changed from full-time jobs to part-time jobs. Quitting from part-time jobs deteriorated mental health gradually and HLFC moderately compared to full-time jobs. | Adults who retire from full time jobs deteriorate in their mental health and functioning. Work is an effective way of social participation for the over 65s. |
| Morelock 2017  US | Older workers healthcare n=437 | Time and place management intervention (management of choice and control processes not only flexible working options) | Workability – competence, health, mental and physical requirements to carry out a job.  Survey data and telephone interviews. | The research team and Modern Medical personnel developed a 30-min learning module. The learning module encouraged greater discussion among managers and employees about making TPM fit requests, or requests to change schedules for a better fit between work and personal responsibilities. The intervention had a positive effect on the relationship between age and workability.  Baseline workability and outcome workability are positively correlated at r = .507 (p < .05), age is negatively correlated with outcome workability, at r = −.143 (p < .05). There was slight evidence that occupation was a factor in workability in this sample. Several main effects (treatment, b = −.451, p < .01; baseline workability, b = 0.436, p < .001), two-way interactions (Treatment × Baseline Workability, b = 0.276, p < .01; age and baseline workability, and b = 0.026, p < .05), and threeway interactions (Treatment × Age Squared × Baseline Workability, b = −0.006, p < .05) have significant effects. | The intervention is promising for older workers with low workability |
| Okamoto 2018  Japan | Aged 60 or older males  N=1288 | Cohort study.  Followed for up to 15 years. Survey samples men and women, followed up every three years, data collected via face to face interviews. National Survey of the Japanese Elderly. Propensity score method of analysis. | Death, cognitive decline via mental status questionnaire, self-reported symptoms of stroke, diabetes | Men in paid employment lived 1.91 years longer (95% confidence interval, CI: 0.70 to 3.11). Difference in onset time p<0.001.  Those in employment had an additional 2.22 years (95% CI: 0.27 to 4.17) before experiencing cognitive decline p=0.003 (unemployed = 7.58 years to onset, while men with employment had 11.20 years). Significantly fewer percentage in employment affected 3% versus 6% p<0.001.  Those in employment had a longer period before the onset of stroke 8.03 years versus 5.84 years p=0.0001. Difference in percentage affected p=0.007 14% versus 20% stroke.  No difference in time to onset (3.96 versus 4.06 years) or percentage affected for diabetes both 23% p=0.878.  Subjective feelings of being in poor health were significantly higher for individuals not in employment (average 17% versus 7%).  Those who were self-employed had longer life expectancy than employees, but adverse diabetes/stroke outcomes. | Being in paid work past the current age of retirement has positive effects on health. |
| Potocnik 2013  European dataset | Retirees and older employees: 2,813 retirees and 1,372 older employees. Average age was 69.79 years, with a range of 50–99 (SD = 7.61). Retirees had a mean of 9.92 (SD = 4.43) years of education. | Cohort study, 2 year follow up: survey of health, ageing and retirement in Europe (SHARE) project – aged 50+ | Engaging in activities Depression  Quality of life | Volunteering or engaging in sporting and social clubs improved quality of life of retirees baseline to follow up. These activities had no impact on older employees. For older employees with low levels of depression though, taking part in political or community organisations was associated with decreased depression at follow up (although those with higher depression at baseline had reduced effect). | Intervention programmes and preventive measures should stimulate engagement in community and leisure activities |
| Stenholm 2014  Finland (data from US) | Aged 65-85 years average age 69 in working group and 74 in retired group.  N=17,844  Those in full time employment, not disabled or those full time retired.  Greater proportion males than females in all full time work groups (61-74%), similar age of retirees. 81-90% White, around 50% high school educated, spread of wealth, around 40% never smoked, around 40% normal weight, around 33-40% at least one disease, 45% of those retired two or more diseases. | Cohort study  Data from US Health and Retirement Study 1992-2010, collected every 2 years. Average length of follow up 5.6 years.  Linear regression analysis. | Self-reported physical functioning (0-10 score) included mobility and activities of daily living | The number of physical functioning difficulties was higher with increasing age, but the increase was significantly smaller while in full-time work than in retirement (test of interaction p=0.002). The number of physical functioning difficulties increased 0.49 (95% CI 0.31 to 0.67) per every 10 years increase in age when in full-time work, and by 0.63 (95% CI 0.54 to 0.72) when in retirement.  The number of physical functioning difficulties was higher amongst women, those with low education or low non-housing financial wealth, both among the full-time workers and retirees. | Physical functioning declines faster in retirement than in full-time work, The absence of chronic diseases and lifestyle-related risks amongst full time workers did not completely explain the differences.  Extending working life may help to maintain physical functioning even among very old adults. |
| Tomioka 2018  Japan | N=6417  Aged over 65 years  Four groups – those not working, those retired during study, those not previously working but acquired new paid work, those continued paid work | Cohort study, three year follow up.  Postal questionnaire.  Mean age at baseline was 72.9 years (range  65–99 years), 47.9% were men and 19.0% reported  having paid work at baseline. | Care needs  Cognitive decline  Activities of daily living | Older men who were not working at baseline but sought work had a decreased likelihood of having long term care (0.41 CI 0.17-0.98) than those retired.  Men who continued working had less likelihood of requiring long term care (OR 0.22 95% CI 0.09-0.54) and cognitive decline (OR 0.69 95% CI 0.09-0.66) than those retired or who started employment.  Men who sought work had less likelihood of decline in activities of daily living (0.42 CI 0.22-0.78) than those retired or continuing to work.  Older women who started working were less likely to require long term care (0.24 CI 0.09-0.66) and have lower risk of decline in activities of daily living (0.39 CI 0.16-0.99) than those retired or continuing to work.  Older women who continued to work had a lower risk of decline in activities of daily living (0.39 95%CI 0.16-0.99), cognitive performance (0.40 CI -.22-0.71) and long term care (0.32 CI 0.15-0.68) than those who retired.  Note: the Crude ORs for those retired had wide Cis crossing 1. | Encouraging older people to stay in the workforce contributes to increasing healthy life expectancy |
| Welsh 2016  Australia | 836 older workers (440 men and 396 women) aged 50–59 years at baseline (2002).  556 (66.5 %) were classified as continuing workers, 192 (22.9 %) as voluntary retirees and 88 (10.5 %) as involuntary retirees. | Cohort study, 9 year follow up.  Household, Income and Labour Dynamics in Australia (HILDA) Survey. Interview data supplemented with postal survey for “sensitive questions”.  Regression analysis | Self-rated physical and mental health, health behaviour: within-person change in self-rated, physical and mental health and one health behaviour (physical activity) at two time points over a nine year follow-up period. | Little difference in health outcomes between those working and those who voluntarily retired. The models showed no evidence of significant difference for self-rated health (p=0.825), physical functioning (p=0.687) or mental health (p=0.123).  However when employment was considered as separate terms of job quality, health outcomes diverged. Compared to voluntary retirees, older workers who had worked in good quality jobs reported marginally better self-rated health (0.14−0.02–0.29); but did not differ in their physical (2.31,−1.09–5.72) or mental health (0.51,−1. 84–2.87). In contrast, older workers who held poor quality jobs for most of the follow-up period declined in their self-rated (−1.13−0.28−–0.02), physical (−4.90, 8.52–−1.29) and mental health (−4.67, 7.69–−1.66) relative to voluntary retirees. Older workers who held poor quality jobs part of the follow-up period did not differ from voluntary retirees in terms of their health. But there was evidence of a linear relationship between length of exposure to poor quality jobs and decline in health outcomes. There was a trend among continuing workers (compared to voluntary retirees) for reduced levels of physical activity over time (p=0.056). | Ensuring older workers have  access to secure jobs which allow for control over work time, skill use and fair rewards is essential if policy goals to boost participation and productivity, as well as reduce the health and care costs of the elderly, are to be met |

**5: Completed quality appraisals**

**Reviews**

| **Author/year** | **1 Focused question** | **2 Right type of papers** | **3 All relevant included** | **4 Quality appraisal** | **5 Results combined reasonable?** | **6 Precision of results** | **7**  **Local population** | **8 Outcomes**  **considered** | **9 Benefits / harms** | **Notes** |
| --- | --- | --- | --- | --- | --- | --- | --- | --- | --- | --- |
| Blok 2011 | Y | Y | Y | NR | Y | Y | NA | Y | NA |  |
| Farrow 2012 | Y | Y | Y | NR | Y | Y | NA | Y | NA |  |

**Randomised controlled trials**

| **Title/ author** | **1 Focused question** | **2 Random assignment** | **3 Ppt accounted for** | **4 Blinded** | **5 Groups similar** | **6 Groups treated same** | **7 Treatment effect size** | **8 Treatment effect precision** | **9 Local context** | **10 Outcomes considered** | **11 Benefits worth costs?** | **Notes** |
| --- | --- | --- | --- | --- | --- | --- | --- | --- | --- | --- | --- | --- |
| Morelock 2017 | Y | Y | Y | N | Y | Y | Y | Y | Y | Y | Y |  |

**Cohort and cross sectional studies**

| **Author/year** | **1**  **Clearly focused issue** | **2**  **Recruitment acceptable** | **3**  **Exposure** | **4**  **Outcome** | **5**  **Confo**  **unders** | **6**  **Follow up** | **7**  **Precision of results** | **8**  **Believe results** | **9 Local help** | **10 Fit** | **11 Prac-tice** | **Notes** |
| --- | --- | --- | --- | --- | --- | --- | --- | --- | --- | --- | --- | --- |
| Anxo 2019 | Y | Y | Y | Y | Y | Y | Y | Y | Y | Y | NA |  |
| Carrino 2018 | Y | Y | Y | Y | Y | Y | Y | Y | Y | Y | NA |  |
| Di Gessa 2017 | Y | Y | Y | Y | Y | Y | Y | Y | Y | Y | NA |  |
| Di Gessa 2018 | Y | Y | Y | Y | Y | Y | Y | Y | Y | Y | NA |  |
| Fujiwara 2016 | Y | Y | Y | Y | Y | Y | Y | Y | Y | Y | NA |  |
| Kajitani 2011 | Y | Y | Y | Y | Y | Y | Y | Y | Y | Y | NA |  |
| Kalousova 2015 | Y | Y | Y | Y | Y | Y | Y | Y | Y | Y | NA |  |
| McDonough 2017 | Y | Y | Y | Y | Y | Y | Y | Y | Y | Y | NA |  |
| Minami 2015 | Y | Y | Y | Y | Y | N | Y | Y | Y | Y | NA | Cross sectional no follow up |
| Okamoto  2018 | Y | Y | Y | Y | Y | Y | Y | Y | Y | Y | NA |  |
| Potocnik 2013 | Y | Y | Y | Y | Y | Y | Y | Y | Y | Y | NA |  |
| Stenholm 2014 | Y | Y | Y | Y | Y | Y | Y | Y | Y | Y | NA |  |
| Tomioka 2018 | Y | Y | Y | Y | Y | Y | Y | Y | Y | Y | NA |  |
| Welsh 2016 | Y | Y | Y | Y | Y | Y | Y | Y | Y | Y | NA |  |
